# Perception-production relations in later development of American English rhotics

**DOI:** 10.1371/journal.pone.0172022

**Published:** 2017-02-16

**Authors:** Tara McAllister Byun, Mark Tiede

**Affiliations:** 1 Department of Communicative Sciences and Disorders, New York University, New York, New York, United States of America; 2 Haskins Laboratories, New Haven, Connecticut, United States of America; University of Akron, UNITED STATES

## Abstract

It is known that some adult listeners have more sharply defined perceptual categories than others, and listeners with more precise auditory targets are also more precise in their production of contrasts. There is additionally evidence that children who have not yet mastered production of a contrast show diminished performance on perceptual measures of the same contrast. To date, however, few studies have investigated developmental perception-production relations using the fine-grained measures typical of adult studies. Existing evidence suggests that perception and production can be closely connected in development, but this relationship may break down as perception and articulation mature at different rates. This study evaluated perception and production of the English /r-w/ contrast in 40 typically-developing children aged 9–14. Perceptual sensitivity was measured with a logistic function fitted over responses in a forced-choice identification task using two synthetic 10-step continua from *rake* to *wake*. Participants also produced rhotic and non-rhotic words. Across participants, there was a significant correlation between perceptual acuity and rhoticity in production, although this effect was only observed for one of two continua tested. These results provide preliminary evidence compatible with the hypothesis that children with a more refined auditory target for a sound also produce that sound more accurately.

## Introduction

### Links between speech perception and production

Recent decades have seen notable additions to a body of research showing that individual variation in speech production is correlated with individual differences in perceptual categorization and discrimination of speech. For example, Perkell *et al*. [1] and Ghosh *et al*. [2] found that adult participants who exhibited greater sensitivity in discriminating intermediate tokens on a /s/-/ʃ/ continuum also showed a larger acoustic contrast between /s/ and /ʃ/ in production. Other evidence of perception-production correlations has been reported by Newman [3], Villacorta *et al*. [4], and Perkell *et al*. [5]. The observed links between perceptual acuity and robustness of contrast in production find a ready account in theoretical and computational models such as the Directions into Velocities of Articulators (DIVA) framework [6, 7]. In the DIVA model, the targets of speech are dynamic regions in auditory-acoustic and somatosensory space. (The auditory targets are considered primary, and they will also constitute the focus of the present paper, although somatosensory targets play an important role as well; see discussion in Ghosh *et al*. [2].) It follows that speakers who identify a narrower region of auditory space as the target for a given speech sound can also be expected to be more precise in their phonetic realization of that sound in contrast with other phonemes.

Support for the sensory targets proposed in the DIVA model has been derived from a range of populations and experimental paradigms (e.g., [8–12]). While most of these studies have made use of adult participants, the DIVA model contains at its core a model of the acquisition of speech (e.g., [13]). Computational modeling of learning in the DIVA framework starts at a point where infants have already identified auditory-acoustic regions representing the phonetic categories of their language by tracking the distributional properties of adult speech (e.g., [14, 15]). In the initial “babbling phase”, the simulated infant engages in semi-random movements of the articulators and learns the mapping between different motor commands and their corresponding sensory consequences [16]. In a subsequent but presumably overlapping “imitation phase”, infants undergo an iterative process to identify and refine the motor commands that produce the closest available match for their auditory encoding of an adult speech model [17]. After executing a motor command, infants use auditory feedback to evaluate the goodness of their output and make adjustments to the motor command in order to produce an output closer to the adult target. Children with more acute perception can be expected to specify a more narrowly defined region of auditory space as the target for a sound, and to undertake more refined motor adjustments to match that target. Thus, the DIVA model predicts that perceptual acuity and degree of distinctness in production should be correlated across speakers in the child population as well as in adults.

In keeping with this prediction, numerous studies have reported that children who exhibit errors in the production of a given contrast also show lower perceptual acuity for that contrast than children who produce it accurately [18–22]. However, most of these studies have used fairly coarse-grained measures of production, e.g. classifying children into groups that either realize or neutralize a contrast on the basis of broad transcription. Relatively few studies have investigated whether the fine details of phonetic production correlate with precise measures of perception in children as they do in adults. Recently, such comparisons have been undertaken in connection with research investigating how children respond to perturbed auditory feedback. Both adults [23, 24] and children [25, 26] have been found to adjust their output to offset such perturbations. The findings emerging from auditory perturbation studies in children have interesting implications for the development of perception-production relations.

Shiller and Rochon [27] examined how 5–7 year-old children responded to a task in which their own speech was synthetically altered by shifting the first formant and played back in near real-time. To investigate the relationship between auditory acuity and compensation in production, Shiller and Rochon [27] measured children's response to F1 perturbation before and after a period of auditory training. Children who completed a perceptual training task targeting the same acoustic dimension (F1) showed a greater degree of compensation in production after the training exercise, while children who received training on an unrelated perceptual contrast showed no change in the magnitude of compensation. The authors interpreted these results as evidence that the children had the capacity to produce greater adjustments in response to perturbation than they had demonstrated at baseline, but first they had to be trained to perceive and/or attend to subtle perceptual differences.

In another recent study of children's ability to compensate for perturbed auditory feedback, Terband *et al*. [28] compared a group of typically developing Dutch-speaking children aged 4–8 and an age-matched group of children with speech sound disorder (SSD). Children's productions of the vowel /e/ in a CVC word context were played back with shifts in the height of the first and second formants. The typically developing children successfully compensated for the perturbation in both F1 and F2. Children with SSD failed to compensate for the shift in either formant, and in the case of F1, they shifted their production in the opposite direction to the typically developing children, exaggerating the magnitude of the perturbation instead of compensating for it. This suggests that children in the SSD group had auditory-perceptual sensitivity adequate to detect the perturbation, but they had difficulty modifying their speech to offset this change. While these results allow for multiple interpretations, one possibility is that the children with SSD had particular difficulty integrating auditory feedback into their existing motor plan to produce an adjustment in the appropriate direction.

These studies highlight an interesting dissociation. In adults, where both speech-motor and perceptual systems are well-established, there appears to be close coupling between perception and production abilities. In children, similar parallels can be observed, but the situation is more complex: both motor and perceptual systems are still undergoing physiological maturation and gaining skill through accumulated experience. It is logically possible for either system to serve as the limiting factor in a given individual or a particular stage in development. That is, a child could perceive a speech target in a relatively adult-like way, but lack the motor skill to realize that target; this is one possible interpretation of the result reported for children with SSD by Terband *et al*. [28]. In a different context, a child speaker could possess adequate motor skill to realize a given target but have an overly broad auditory-perceptual representation of that target, with the consequence that they do not receive the error feedback that would lead them to update and refine their motor plan [27].

Shiller and Rochon [27] suggest that a more complete understanding of perception-production relations over the course of development represents a promising direction “to better grasp the factors underlying the enormous variability in speech production ability observed among children” (p. 1308). Understanding when there is likely to be developmental dissociation between perception and production, and whether perception or production will represent the limiting factor, would be particularly valuable in guiding the clinical management of childhood speech sound delay or disorder. However, it is not clear that we can make valid predictions in this regard. In both perception and production, children undergo profound changes at the earliest stages, followed by gradual refinement over an extended period. The development of production relative to perceptual skills may show variation across different speech targets, speakers, and stages of development. The study reported here aimed to extend our understanding of developmental perception-production relations by focusing on a relatively circumscribed context: the later stages of development of North American English rhotics.

### Perception and production of North American English /ɹ/

The North American English rhotic /ɹ/ is a late-emerging sound, not reaching mastery level in the population until over eight years of age [29]. Among English-acquiring children with a history of speech delay or disorder, an estimated 30% continue to exhibit residual errors affecting rhotic sounds at 9 years of age, and roughly 9% show these errors from 12–18 years [30]. The difficulty that children experience in acquiring /ɹ/ is thought to be attributable at least in part to the complexity of the articulatory configuration used to produce the sound [31]. While most speech sounds are produced with only one major constriction or narrowing of the vocal tract, articulatory descriptions identify two major lingual constrictions that make a crucial contribution to the acoustic properties of /ɹ/ [31–33]. Imaging studies have demonstrated that speakers use a range of vocal tract configurations to produce /ɹ/, including the well-known *retroflex* and *bunched* categories and various intermediate tongue shapes [34, 35]. Furthermore, it is common for a single speaker to use multiple tongue configurations across different coarticulatory contexts [8, 36, 37]. Guenther *et al*. [8] argued that this contextual variability in articulation promotes consistency in attaining the acoustic signature that differentiates /ɹ/ from other sonorant phonemes. The acoustic hallmark of rhoticity is a lowered third formant, F3 [34, 38]. In English /ɹ/, this lowered F3 coincides with a relatively high second formant, yielding a small F3-F2 distance [39].

Several previous studies have investigated perception-production relations in English rhotics, including in the developmental context [11, 18, 40]. Hoffman *et al*. [18] compared 6-year-old children who replaced /ɹ/ targets with the glide /w/ against a group of age-matched controls. In a task of classifying intermediate stimuli on a 7-step synthetic continuum from *ray* to *way*, children who exhibited /ɹ/ gliding also showed a significantly flatter identification function than control participants, indicating reduced consistency in perceptual categorization. Idemaru and Holt [40] examined the emergence of primary and secondary cues to rhoticity in the perception and production of /ɹ/ and /l/ in children between 4 and 8.5 years of age. For adult listeners, F3 lowering is the primary cue to rhoticity, with F2 height contributing as a secondary cue. Based on the frequency and reliability of cues in the input, Idemaru and Holt [40] hypothesized that F3 would play a dominant role in children's early perception and production of /ɹ/ versus /l/. They found support for this prediction: children's utilization of the secondary F2 cue appeared to lag behind their control of F3 in both perception and production. However, they found "no systematic relationship between children's production and perception of /l/ and /r/" (p. 4243). Young children's articulatory limitations were cited as one possible explanation for this lack of correlation. McGowan *et al*. [41], observing that F2 height increased with age in toddlers acquiring /ɹ/, suggested that some child speakers lack the articulatory skill necessary to lower F3 to adult-like levels, and instead rely on decreased F3—F2 distance to mark the rhotic category. At the same time, young children produce /l/ with an unusually high F2 because of their use of light /l/ instead of the articulatorily more complex dark /l/ (see detailed discussion in Lin and Demuth [42]). If these production-oriented factors do play a prominent role in determining F2 height in both /ɹ/ and /l/, it could mask any influence of perception, accounting for Idemaru and Holt's [40] failure to find a systematic relationship between perception and production of the F2 cue.

The present study investigated whether a correlation between fine-grained measures of rhotic perception and production would emerge in an older sample of children. The 9–15 year age range was targeted because previous evidence suggests that ongoing maturational refinement can be expected in both perception (e.g., [43]) and production (e.g., [44]). At the same time, this older sample should be less influenced by the articulatory limitations that were posited to have masked perception-production correlations in Idemaru and Holt [40]. If perceptual acuity accounts for variance in rhoticity above and beyond any effect of age, this would support the hypothesis that children’s rhotic productions are influenced by the specificity of an auditory target, as described for adults by Ghosh *et al*. [2] and similar studies.

A secondary purpose of this study was clinical in nature: it serves as a normative sample against which perception in older children with residual speech errors affecting rhotics can be assessed. In North America, children with reduced rhoticity are often referred to a speech-language pathologist for treatment to encourage a more typical production of /ɹ/. However, speech-language pathologists describe the /ɹ/ sound as particularly challenging to remediate, and many children are discharged from intervention with their errors uncorrected [45]. Correction of rhotic misproduction is customarily approached as an articulatory learning task (e.g., [46]). If perceptual limitations are found to play a role (as suggested by [47]), a modified approach to intervention may be indicated. Recent work has specifically highlighted the clinical need for tools that can provide a fine-grained measure of rhotic perception in this population [48].

For clarity, below we present the production experiment first, followed by the perception experiment, and finally an examination of correlations between the two. However, both perception and production tasks were administered to each participant in a single session approximately 1.5 hours in duration. The order of presentation of subtasks was constant across participants, as follows: (1) 40 production trials (target words *raid*, *wade*); (2) 40 production trials (target words *trade*, *braid*, not analyzed here); (3) 40 identification trials (female speaker continuum); (4) Number Repetition subtest of the *Clinical Evaluation of Language Fundamentals—4th Edition* [49], to measure digit span; (5) 40 identification trials (female speaker continuum); (6) extended break; (7) 40 identification trials (male speaker continuum); (8) 40 production trials (target words *bird*, *bud*); (9) 40 production trials (target words *bard*, *beard*, not analyzed below); (10) 40 identification trials (male speaker continuum). Written consent to participate in the study was obtained from both participants and their parent/guardian. The study protocol, including this consent process, was approved by the New York University Committee on Activities Involving Human Subjects (protocol #13_9382).

## Materials and methods: /ɹ/ Production

### Participants

Forty native speakers of English were recruited from schools and community centers in New York, NY. Participants were eligible for inclusion only if parent report indicated no major history of neurobehavioral, speech-language, or hearing impairment. They were additionally required to pass a hearing screening at 500, 1000, 2000, and 4000 Hz at 20 dB HL. Because a substantial proportion of this urban population is multilingual, all native speakers of American English were eligible for inclusion in the study; speaking other languages was not an exclusionary criterion. Thirty-four out of 40 participants were reported to hear mostly or only English in the home, five children were described as hearing English and another language in roughly equal amounts, and one child was described as hearing mostly Spanish in the home. Ten out of 40 participants were described as proficient speakers of a language other than English. Participants additionally were not screened for the rhoticity of their home dialect. (It is acknowledged that from a measurement standpoint, a homogeneous sample of monolingual, monodialectal speakers of rhotic American English would be preferable to the more diverse sample actually admitted. Recall, though, that this study also serves as a normative comparison for children receiving treatment for rhotic misarticulation, and it was necessary to use the same demographic criteria for inclusion/exclusion across the two studies. Excluding children from a treatment study on the basis of the language or dialect spoken in the home would be ethically problematic, because it would disproportionately affect children from linguistically and culturally diverse backgrounds.) However, data were discarded post hoc from two participants with missing perceptual results, two participants who represented outliers on production measures, and three participants who represented outliers on perceptual measures. The manner in which these exclusions were determined is described in detail in the relevant sections below.

The group of 40 participants initially enrolled in the study had a median age of 139 months (11;6), with a range from 108 to 177 months (9;0–14;8). After exclusions, there were 33 participants, of whom 16 were female and 17 were male. The final group had a median age of 142 months (11;9). The age range was similar across male and female subgroups (108–176 months and 110–177 months, respectively). Unless otherwise indicated, all data reported below reflect the sample of 33 participants after exclusions.

### Production task

The primary production measure was structured to conform to the task design followed in Ghosh *et al*. [2]. The measure elicited repeated productions of words containing rhotic and non-rhotic sounds. All word targets were embedded in the carrier phrase "Say X for me." One subtask elicited /ɹ/ and /w/ in onset position in the word pair *raid*-*wade*, and a second subtask elicited the rhotic vowel /ɝ/ and the non-rhotic vowel /ʌ/ in the word pair *bird*-*bud*. (These targets were selected because *raid-wade* provides the closest parallel for the perceptual stimuli *rake-wake*, while the relatively long duration of the syllabic /ɝ/ in *bird* provides an opportunity for participants to produce a more hyperarticulated rhotic.) Following Ghosh *et al*. [2], within each subtask, each word in a pair was elicited ten times in a clear speech condition and ten times in a casual speech condition. Productions were blocked by word and speaking condition (e.g., *raid* x 10 careful, *wade* x 10 careful, *wade* x 10 casual, *raid* x 10 casual), and order of elicitation within each subtask was counterbalanced across participants. In the clear speech condition, participants were instructed to speak “in a clear voice, as if you were talking to someone who can’t understand English very well,” and in the casual condition, they were instructed to speak “in a regular voice, as if you were just having a normal conversation with a friend.” (Different speech conditions are intended to elicit different degrees of hyper- or hypoarticulation, which could show different degrees of association with perceptual acuity. Due to time constraints in the context of child participants, a third “fast” speech condition elicited by Ghosh *et al*. [2] was not included in this study.) The written target word within the carrier phrase context was presented on the computer screen during all trials, along with a pictorial cue reminding the participant of the desired speech condition in a given block. The research assistant administering the task provided verbal models and reminder cues to elicit the desired speech condition.

All recordings were elicited in a soundproofed booth. Participants spoke into a table-mounted Shure SM48 microphone and were monitored to maintain a 5-inch mouth-to-microphone distance. Recordings were obtained with a 44 kHz sampling rate and 16-bit encoding.

### Measurement of production

The primary acoustic measure of rhoticity in production was the distance between the second and third formants (F3-F2), where a lower F3-F2 distance correlates with a more strongly rhotic /ɹ/ or /ɝ/. F3-F2 distance was used instead of raw F3 because the latter is highly sensitive to the length of the speaker's vocal tract, which in turn varies with age, gender, and height. F3-F2 distance provides a partial correction for vocal-tract dependent differences in raw formant height and has been suggested as a particularly valuable option in measuring children's /ɹ/ sounds [41, 44, 50].

Praat acoustic analysis software [51] was used to obtain formant frequency measures in each target sound. To measure formants, trained student assistants listened to the audio record of each utterance while viewing a spectrogram with automatic LPC tracking of the first five formants. The number of LPC coefficients was selected on an individual basis for each participant [52] via inspection by the first author. After identifying the portion of the acoustic record corresponding with the target sound (/ɹ/, /w/, /ɝ/, or /ʌ/), the student placed a cursor at a point that was judged to represent the minimum height of F3 in the target interval. Points that appeared as outliers relative to adjacent points in the automated formant track were avoided. This process was repeated for all target words in all probe files. Finally, an automated algorithm [53] was used to extract measurements of the first three formants from the selected point in each word; only F2 and F3 were used in the analyses reported below. To assess the reliability of these initial measures, 10% of files were re-measured by different research assistants. An intraclass correlation (ICC) with single random raters was calculated to be .95, indicating strong interrater agreement.

For data cleaning purposes, means, medians, and standard deviations across all repetitions of each rhotic target (*bird*, *raid*) were plotted for each participant. Any values that stood out on visual inspection were re-measured by an experienced graduate student to identify possible errors in formant tracking. A total of 17 files were checked in this way. The two participants whose median values after remeasurement continued to fall more than 2 median absolute deviations (MAD; [54]) from the median across participants were excluded from analyses of production data.

### Results of production task

**Fig 1:**
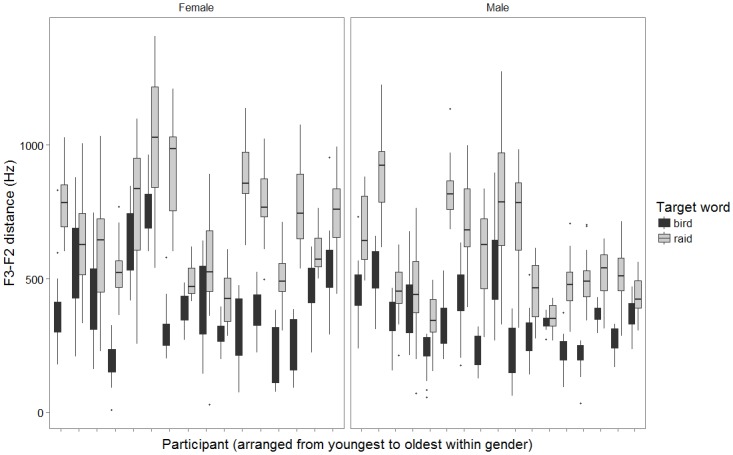
Distributions of acoustic values (F3-F2 distance) measured across up to 20 repetitions of each word (*bird*, *raid*) for each speaker. Speakers are subdivided by gender and rank-ordered by age. Productions are pooled across careful and casual speech conditions.

**Fig 2:**
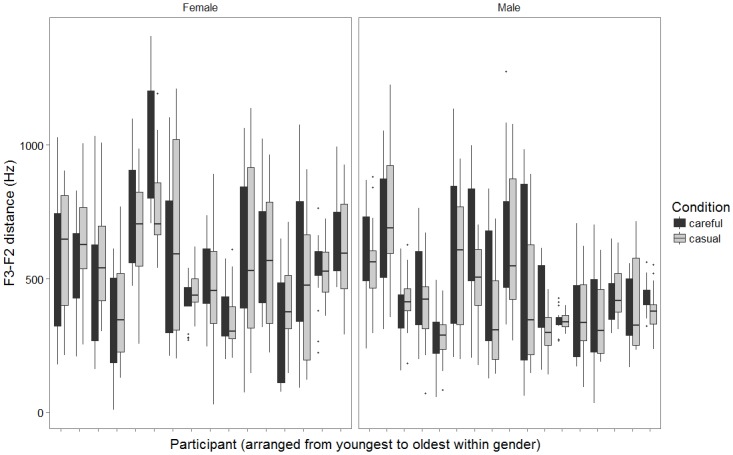
Distributions of acoustic values (F3-F2 distance) measured across up to 20 productions in each speech condition (careful, casual) for each speaker. Speakers are subdivided by gender and rank-ordered by age. Productions are pooled across the target words *bird* and *raid*.

To give a general sense of within and across-subject variability, Figs 1 and 2 use boxplots to display the distribution of F3-F2 distance values measured across up to 20 repetitions of rhotic target words for each of the 33 included speakers. Because formant heights can covary with factors such as age and gender, the speakers represented in Figs 1 and 2 have been partitioned by gender and rank-ordered by age in months. In Fig 1, measurements from syllabic /ɝ/ in *bird* and onset /ɹ/ in *raid* are displayed separately, while the careful and casual speech conditions are pooled. In Fig 2, the reverse is true.

Two linear mixed models were used to examine how F3-F2 varied in association with speaker properties and task conditions, respectively. The first model considered fixed effects of speaker age and gender, with a random by-subject intercept. The interaction between age and gender was not included in the model because a likelihood ratio test indicated no significant difference between the models with and without the interaction. There was a significant effect of gender, with male speakers showing a lower F3-F2 distance than females (β = -97.64, SE = 43.29, p = 0.03). The effect of age was not significant (β = -1.99, SE = 1.02, p = 0.06). The second model examined fixed effects of word (*bird*, *raid*) and speech condition (careful, casual). Again, no interaction term was included because a likelihood ratio test indicated that the crossed model did not differ significantly from the simple model. A by-subject random intercept was included to reflect the nested structure of the data. The main effect of word was significant (β = 261.66, SE = 8.47, p < 0.01), but speech condition was not (β = -14.99, SE = 8.47, p = 0.08). As a consequence, the careful and casual speech conditions will be pooled in all subsequent analyses to increase experimental power.

## Materials and methods: /ɹ/ Perception

### Participants

All 40 participants who enrolled in the study completed the perception task, but there were five post-hoc exclusions, as noted above. In two cases, experimenter error resulted in perceptual responses that were not correctly registered and could not be recovered. An additional criterion was applied to exclude participants whose perceptual boundary width fell more than two MAD away from the group median. A total of three participants were excluded due to boundary width scores that represented upper outliers, where a high boundary width indicates an inconsistent response pattern; there were no lower outliers. These exclusions were made because it could not be ruled out that the high level of inconsistency exhibited by these participants reflected lack of attention to the task. (Data from the three participants who were excluded on the basis of low perceptual acuity were qualitatively examined to determine if these individuals represented outliers with respect to any other parameters. Measures of median F3-F2 distance from the three exclusions would fall solidly within the interquartile range for the rest of the group. Likewise, these excluded participants did not represent outliers with respect to age or digit span.)

### Perception task

In a forced-choice button-press task, participants identified speech stimuli from synthetically modified continua as instances of the words *rake* or *wake*. Two separate 10-step acoustic continua from /ɹ/ (*rake*) to /w/ (*rake*) were synthesized using naturally produced tokens of the words, elicited in isolation from one 10-year-old female and one 8-year-old male speaker. Child speech stimuli were used with the rationale that this would provide a more appropriate point of comparison for productions elicited from the child and adolescent participants in the present study. Each *rake* token was transformed into a 10-step continuum by morphing 13 LPC coefficients calculated at 20 ms intervals between the values for the *rake* and *wake* endpoints while holding the residual (pitch and release noise) constant. The first step in the continuum was the resynthesized version of the naturally produced /ɹ/, with no morphing; this step was used in training but not in experimental trials. The /ɹ/ in *rake* was aligned by the onset of voicing with a naturally produced token of *wake* elicited from the same speaker. LPC coefficients, gain, and associated residuals were calculated over the voiced portion of the aligned utterances. For each successive step, the weighted average between the coefficients and gain was calculated, and the token was resynthesized using the /ɹ/ residual. Average RMS intensity was scaled to 70% of the full-scale range for each sound file. Table 1 reports formant frequencies associated with each step in the continua synthesized from the female and male speaker samples.

**Table 1:** Formant frequencies associated with original tokens and synthesized continuum steps for female and male speaker stimuli. Only steps 2–10 were presented in experimental trials. All measures reported in Hertz.

	Female Continuum				Male Continuum			
Transition Midpoint (80 ms)	F1	F2	F3	F3-F2	F1	F2	F3	F3-F2
**/r/ (original)**	455	1127	1814	687	410	1339	2059	720
**Step 1**	452	1120	1837	717	434	1522	2349	827
**Step 2**	452	1118	1839	721	434	1517	2349	832
**Step 3**	452	1113	1844	731	435	1502	2350	848
**Step 4**	453	1104	1852	748	436	1473	2347	874
**Step 5**	453	1094	1863	769	439	1432	2341	909
**Step 6**	454	1082	1857	775	445	1395	2338	943
**Step 7**	456	1071	1859	788	449	1373	2340	967
**Step 8**	456	1062	1894	832	447	1351	2346	995
**Step 9**	459	1057	1912	855	444	1323	2350	1027
**Step 10**	461	1054	1931	877	448	1284	2351	1067
**/w/ (original)**	441	683	1775	1092	365	810	1902	1092

Feedback regarding perceptual naturalness and clarity was elicited from child and adult pilot participants and was used to guide a series of refinements to the synthetic continua. Because the coda stop burst was reported to create a nonspeech-like effect over the course of repeated presentations, the burst was excised, re-normalized to a lower intensity (55 dB), and re-spliced at the zero crossing before the original burst. Second, a schwa-like onglide was perceived at the start of both continua, possibly because the child speakers who contributed the stimuli were extending the duration of the /ɹ/ in their efforts to speak clearly. To eliminate this percept, the first 0.048 ms was excised from each token. Following these adjustments, the continua were judged to be sufficiently perceptually natural.

All perception stimuli were presented via Sennheiser headphones within the soundproofed booth, with the playback volume set to roughly 70% of maximum. For robustness over brief lapses in attention, each trial presented a single stimulus recording two times with a 500-ms interstimulus interval. Participants were instructed to indicate which word they heard by pressing a button labeled with a picture and a written word. For each continuum, participants initially completed a practice block in which the /ɹ/ and /w/ endpoint stimuli were presented four times each in random order. This practice block allowed participants to become familiar with the dual presentation of each stimulus and the button-press response. All participants successfully completed this block. During the main experimental trials, the nine steps within a continuum were presented eight times each in random order (total *n* = 72 trials per continuum), with a break halfway through. As noted above, the two continua were presented in a fixed order: male first, then female. Although a counterbalanced order of presentation would ordinarily be preferable, a fixed order was favored because of the dual role of this study as a normative reference for a concurrent clinical study of children receiving intervention for rhotic speech errors. Because of the small sample size of that study (*n* = 14), obtaining interpretable data from all participants was the most important priority. A fixed presentation order was adopted with the reasoning that it would make it easier to identify and control for any effect of order of presentation.

After data were collected and results were analyzed, it was determined that participants' responses to the male and female speaker continua differed significantly with respect to some measures, discussed in detail below. To better understand these differences, post hoc data collection was undertaken from adult listeners. A sample of 31 self-reported native English speakers were recruited through Amazon Mechanical Turk to complete an online version of the perceptual identification tasks, implemented using the Experigen platform for experiment presentation [55]. After a brief training using the endpoint stimuli, participants heard each stimulus eight times in random order and classified each item as *rake* or *wake*. As in the lab setting, the two continua were presented in a fixed order, starting with the female speaker.

The online protocol differed from the lab-based experiment in two major ways. First, each identification block was followed by a block in which participants were asked to rate the perceptual goodness of each stimulus. In this block, stimuli were again presented eight times each in random order, and the listener rated each trial by clicking on a visual analog scale with endpoints “the w sound” and “the r sound.” Use of a visual analog scale task was intended to elicit a gradient measure of the perceived rhoticity of each token (see, e.g., [56]). The second major difference was that the online data collection setting is less controlled with respect to factors such as the level of background noise or the quality of individual listeners’ audio equipment. Further, we asked participants to report language/dialect background and hearing status and to affirm that they were wearing headphones, but we cannot independently confirm the validity of these responses. The results of the post hoc study will be interpreted with the awareness that online data collection is inherently noisier than lab-based studies, but that a meaningful signal can still be derived by averaging across multiple participants (see discussion in McAllister Byun *et al*. [57]). Outliers were excluded in the same manner described for child participants: individuals for whom either of the two perceptual outcome measures described below fell more than two MAD from the group median were excluded, with the reasoning that these unusual response patterns could potentially reflect a lack of attention to the task. Three participants were excluded in this way, leaving a total of 28. Results from adult participants recruited online are reported below for comparison with results from child participants in the lab setting.

### Measurement of perception

**Fig 3:**
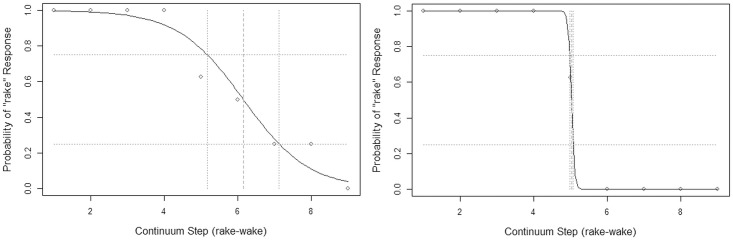
Sample response data and best-fit logistic functions (female speaker continuum). Left panel represents an 11;0-year-old female with a boundary location of 6.14 and a boundary width of 1.95. Right panel represents a 10;6-year-old female with a boundary location of 5.0 and a boundary width of 0.0.

To evaluate performance on the perceptual identification task, a logistic function was fitted over the number of *rake* responses at each step in the continuum, calculated separately for male and female speaker continua for each individual. Two metrics were derived from the fitted function. “Boundary location” represents the point on the continuum where the fitted logistic function reached its 50% probability point. “Boundary width” represents the distance in continuum steps between the 25% and 75% probability points on the fitted logistic function. A wider boundary region, which corresponds to a flatter slope of the fitted logistic function, indicates that a listener is less consistent in categorizing individual stimuli. This, in turn, has been interpreted as evidence that the phoneme categories in question are represented in a less precise or overlapping manner [43, 58, 59]. Previous research has shown that boundary width can provide a fine-grained metric of perceptual acuity, reflecting ongoing refinement with age in children up to 12 years or beyond [43]. As a result, the analyses and discussion that follow will focus more on boundary width than boundary location. Samples of logistic functions fitted to actual participant data, as well as the boundary location and boundary width values derived in each case, are provided in Fig 3.

If a listener was 100% consistent in identifying all points on the continuum except for one boundary point, the boundary width was operationalized as 0.0. This created a ceiling effect that tended to obscure distinctions among the highest-performing participants; we discuss this limitation in greater detail below.

### Results of perception task

This section reports descriptive statistics and correlations for measures of perceptual boundary width and location from both female and male speaker continua. We begin by examining whether results from child participants in the lab setting differed significantly from adult listeners recruited online, followed by an examination of differences between the two continua (female and male speaker) in both groups. We then examine whether boundary width on one continuum correlates significantly with boundary width on the other. Finally, we evaluate whether perceptual boundary width is significantly associated with age or digit span, a measure of working memory. Due to limitations of the data obtained from adult listeners online, these relationships were examined for child participants only.

**Fig 4:**
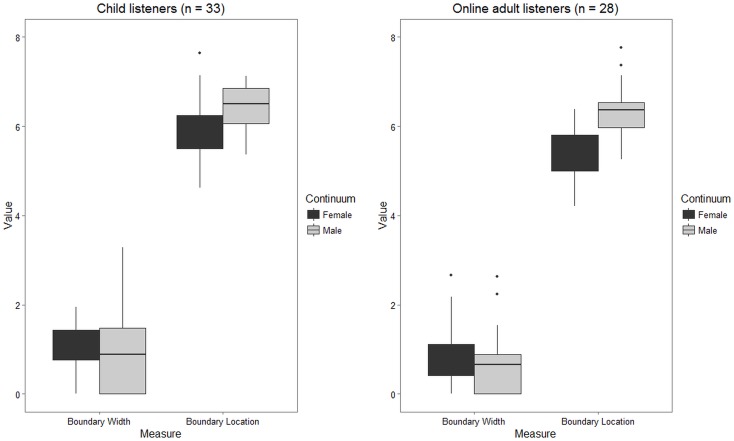
Boxplots of boundary width and location values for both male and female speaker continua. Left panel represents child listeners in the lab setting. Right panel represents adult listeners recruited online.

The boxplots in Fig 4 represent the distribution of boundary width and boundary location values measured in the 33 child participants who were included in our final analyses, along with data from the 28 adult listeners included in the post-hoc norming sample. Both child and adult groups showed considerable variability for both measures, but visual inspection of Fig 4 is suggestive of broad agreement between the lab-based child sample and the online adult sample. The two groups were not found to differ significantly with respect to boundary width for the female speaker continuum (*t* = 1.1, *df* = 51.9, *p* = 0.28), boundary width for the male speaker continuum (*t* = 1.42, *df* = 58.98, *p* = 0.16), or boundary location for the male speaker continuum (*t* = 0.8, *df* = 56.37, *p* = 0.43). However, they did differ with respect to boundary location for the female speaker continuum (*t* = 3.59, *df* = 58.09, *p* < 0.01), with the online adult listeners showing a significantly lower boundary—that is, they were less likely to classify intermediate tokens as instances of *rake*. For both child and adult listener groups, the male speaker continuum showed a significantly higher boundary location than the female continuum. The difference between continua with respect to boundary width was not significant for either group. However, in both groups, more participants achieved the minimum possible boundary width (indicating ceiling-level perceptual acuity) for the male speaker continuum than the female speaker continuum. Descriptive and inferential statistics for these comparisons are summarized in Table 2.

**Table 2:** Descriptive and inferential statistics for both male and female speaker continua.

		Female speaker continuum		Male speaker continuum		Comparison of continua		
Group	Boundary measure	Median (MAD)	N of zeroes	Median (MAD)	N of zeroes	t-value	DF	p-value
Child	Width	0.98 (0.71)	4	0.88 (0.97)	9	0.2	32	0.84
	Location	5.88 (0.83)	N/A	6.5 (0.93)	N/A	-4.32	32	< 0.01
Adult	Width	0.83 (0.44)	7	0.65 (0.87)	9	1.1	27	0.28
	Location	5.25 (0.39)	N/A	6.37 (0.58)	N/A	-7.68	27	< 0.01

Across individuals in the adult group, there was a significant correlation between boundary width measured with the female continuum and boundary width measured with the male continuum (ρ = 0.42, *p* = 0.03). That is, individuals who showed a high level of consistency when classifying stimuli from the female speaker continuum were also likely to show a high level of consistency for the male speaker continuum. Unexpectedly, these measures were not significantly correlated across participants in the child group (ρ = 0.003, *p* = 0.99). Boundary location on the female continuum did not correlate significantly with boundary location on the male continuum for either adults (ρ = 0.33, *p* = 0.09) or children (ρ = 0.26, *p* = 0.15).

Previous studies (e.g., [43]) have shown that categorical perception curves become steeper, and boundary width correspondingly narrower, with increasing age in late childhood. For child participants in the present study, the correlation between age and perceptual boundary width was small but significant for the male speaker continuum (ρ = -0.37, *p* = 0.03), with the negative coefficient indicating that younger children exhibited wider boundary regions than older children, suggesting lower perceptual acuity. The correlation between boundary width and age was not significant for the female speaker continuum (ρ = -0.16, *p* = 0.36).

Although perceptual identification tasks are thought to pose relatively modest demands on working memory [60, 61], the consistency with which a listener responds to speech tokens on a continuum could still be influenced by that listener's ability to hold perceptually encoded traces in short-term memory. To investigate this possibility in our child participants, we additionally examined the correlation between perceptual boundary width and a measure of verbal working memory, scaled score on the Number Repetition subtest of the *Clinical Evaluation of Language Fundamentals—4th Edition* [49]. The observed scaled scores were sufficiently dispersed to examine this correlation (range = 7–17, median 11). These correlations were not significant for either the female speaker continuum (ρ = -0.17, *p* = 0.34) or the male speaker continuum (ρ = -0.22, *p* = 0.22).

## Perception-production relationship

### Analyses

Our main research question asked whether the acoustic characteristics of rhotic sounds produced by children aged 9–15 were significantly correlated with their perceptual acuity in categorizing tokens on synthetic /ɹ/-/w/ continua. In the preceding sections, we saw that our primary acoustic measure, F3-F2 distance, was significantly associated with both the gender of the speaker and the identity of the target word (*bird* versus *raid*). Our measure of perceptual acuity, boundary width, was significantly associated with age for one of the two stimulus continua tested. To understand the relationship between perceptual acuity and production distinctness for children's rhotic sounds, we need to know if boundary width accounts for variance in production above and beyond any variance associated with these additional variables. The perception-production relationship was examined using linear mixed models with median F3-F2 distance as the dependent variable. Because participants showed significantly different behavior in response to the female versus male speaker continua, the perception-production relationship was examined in two separate regressions. In addition to our primary predictor of interest, perceptual boundary width, each model included the age and gender of the participant and the identity of the target word (*bird* or *raid*). A random intercept for participant was included to account for speaker-level variation not captured by the fixed effects. A by-word random slope was examined but not included because it prevented model convergence.

### Results relating production and perception

**Fig 5:**
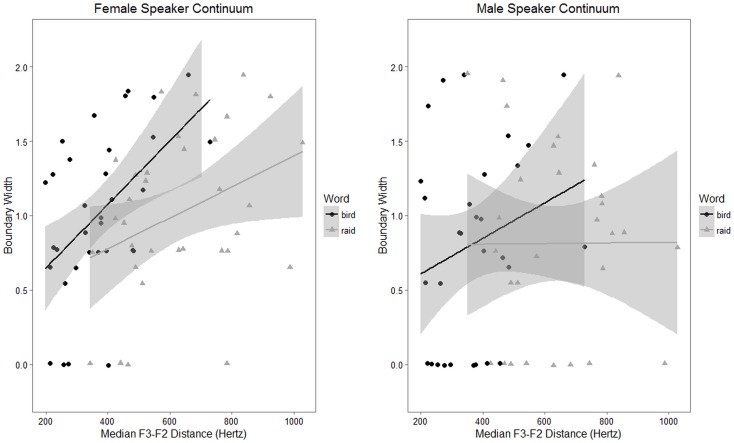
Scatterplots representing boundary width versus F3-F2 distance, partitioned by word. Shaded band represents a 95% confidence interval around the best-fit line.

The results of the mixed model revealed that F3-F2 distance was significantly associated with perceptual boundary width as measured using the female speaker continuum (*β* = 95.85, *SE* = 44.85, *p* = 0.04). Age in months and gender did not emerge as significant predictors in this model (age: *β* = -1.72, *SE* = 0.98, *p* = 0.09; gender: *β* = -39.55, *SE* = 47.99, *p* = 0.42), but the effect of word was strongly significant (*β* = 258.09, *SE* = 29.48, *p* < 0.001). In the regression that examined perceptual acuity as measured with the male speaker continuum, word was found to be a significant predictor of F3-F2 distance (*β* = 258.09, *SE* = 29.48, *p* < 0.001), but age, gender, and perceptual boundary width did not meet the criterion for significance (age: *β* = -2.14, *SE* = 1.1, *p* = 0.06; gender: *β* = -91.06, *SE* = 44.46, *p* = 0.05; boundary width: *β* = -7.36, *SE* = 28.46, *p* = 0.8). We additionally considered models that included first-order interactions of all other predictors with perceptual boundary width. However, because likelihood ratio tests indicated no significant differences between the models with and without interactions, the simpler models were adopted. Scatterplots and best-fit lines for these data are presented in Fig 5.

## Discussion

### Later stages of development of rhotic perception/production

The results of the production study indicated that the syllabic rhotic /ɝ/ was produced with significantly lower F3-F2 distance than the onset rhotic /ɹ/. This finding is consistent with previous empirical research [44, 50] and is also a phonetically sensible result, since the longer duration of the syllabic rhotic gives the speaker time to reach a more extreme articulatory configuration. Previous research (e.g., [44]) has reported a decrease in F3-F2 distance in rhotic production over the course of maturation. In the present sample of children aged 9–15, the association between age and F3-F2 distance was not significant, although the p-value of .06 suggested that this association might be found significant in a larger sample with similar properties.

In a slightly younger group of children (ages 6–12), Hazan and Barrett [43] found a significant association between age and boundary width in a perceptual identification task involving /s/ and /ʃ/. It was thus hypothesized that the present study would reveal a significant negative correlation between age in months and perceptual boundary width, which decreases as perception becomes more acute. For child participants in the present study, the correlation between age and perceptual boundary width was small but significant for the male speaker continuum (ρ = -0.37, p = 0.03), with the negative coefficient indicating that younger children exhibited larger (less acute) boundary widths than older children. The correlation between boundary width and age was not significant for the female speaker continuum (ρ = -0.16, p = 0.36). This finding will be considered in greater detail below in conjunction with our discussion of asymmetries in perception-production relations.

In total, measures of rhotic perception and production tended to vary with age in the direction expected on the basis of previous research. The observed associations were weak and variable, however, suggesting that age may make a relatively minor contribution to individual variation in perception and production in this late stage of maturation.

Finally, we investigated the possibility that individual differences in working memory might account for differences in the consistency with which children identify perceptually ambiguous stimuli. Although participants in the present study varied widely in their scores on a working memory measure, there was no significant association between this measure (digit span) and perceptual boundary width.

### Perception-production relations in rhotic development

The primary objective of this study was to investigate the relationship between perception and production of North American English rhotic sounds in late childhood and early adolescence. The present experiment did yield evidence for an association between rhoticity in production and perceptual acuity for rhotics, but the significance of this relationship depended on which stimuli were used to measure perception. For the female speaker continuum, perceptual boundary width was a significant predictor of median F3-F2 distance, as was the identity of the target word. For the male speaker continuum, word was the only fully significant predictor of median F3-F2 distance.

In short, the hypothesis of an association between perceptual acuity and production distinctness in later stages of rhotic acquisition was supported by the results obtained using the female speaker continuum, but not by the results obtained using the male speaker continuum. Several interpretations are available in connection with this pattern of observations. One possibility is that the two continua provide equally valid measures of perceptual acuity, and any difference in how the continua relate to production measures is a consequence of chance fluctuations. This would suggest that a perception-production relation exists, but is not very robust, for the age group and speech targets studied here. A second possibility is that the two stimulus continua have distinct properties that cause them to differ in how they elicit measures of perceptual acuity. A final possibility is that the difference could be an artifact of order of task administration, since all participants completed the male speaker continuum after the female speaker continuum. The latter two possibilities, which pertain more to experimental design than to the robustness of perception-production relations in the target population, are considered in greater detail below.

**Fig 6:**
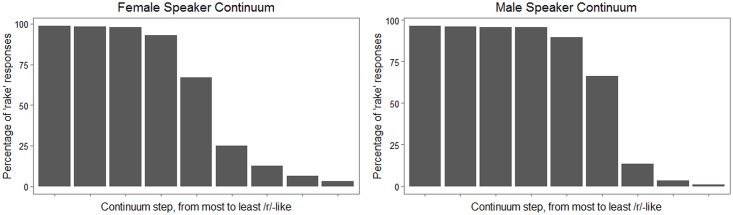
Responses to female and male speaker continua from adults recruited online: Percentage of trials classified as *rake* for each stimulus, pooled across raters.

**Fig 7:**
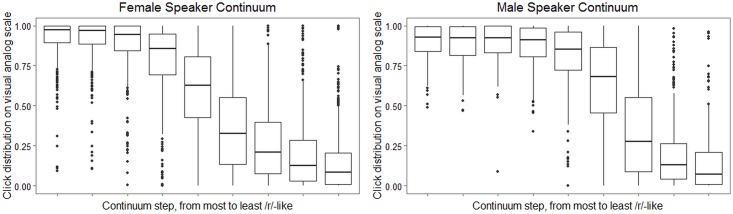
Responses to female and male speaker continua from adults recruited online: Distribution of clicks on a visual analog scale for each token, where 0.0 = “the w sound” and 1.0 = “the r sound”.

Figs 6 and 7, which plot ratings obtained from adult listeners recruited online, strongly support the hypothesis that the two continua do elicit meaningfully different patterns of perceptual response. In both figures, the left-hand plot reflects data from the female speaker continuum, and the right-hand plot shows data from the male speaker continuum. The plots in Fig 6 represent the proportion of times a particular stimulus was identified as the word *rake*, pooled across listeners. The plots in Fig 7 show the distribution of all listeners’ clicks on a visual analog scale with endpoints labeled “the w sound” and “the r sound.” (Recall that adults recruited online were engaged in this additional task, which was not administered to child participants, as a way to obtain more detailed information about the perceptual properties of the stimulus continua.) In Fig 6, it is noteworthy that for the female speaker continuum, listeners show virtually 100% agreement in selecting the *rake* label for the first three continuum steps, while for the male speaker continuum, the first four continuum steps show this same level of agreement. The median click locations in Fig 7 corroborate the impression that the male speaker continuum has a narrower boundary region than the female speaker continuum. Furthermore, in Fig 7, even the extreme *rake*-like tokens for the female speaker continuum show considerable scatter into the far end of the scale representing “the w sound.” In summary, results from online adult listeners suggest that the female speaker continuum was more successful than the male speaker continuum in creating perceptual ambiguity.

The differences that emerge in adult listener data are corroborated to some extent by examination of the acoustic properties of the two continua (see Table 1). These show that from step 1 (the copy-synthesized “rake” token) to step 10 (the extreme synthesized “wake” token), differences in formants F1, F2, F3 for the female speaker continuum were 9 Hz, -66 Hz, and 94 Hz, respectively. For the male speaker continuum, the corresponding differences were 14 Hz, -238 Hz, and 2 Hz. This indicates that the approach of interpolating and resynthesizing from LPC coefficients using the original /r/ residual at each step was less successful for the male continuum. It is possible that because the F3-F2 percept was driven almost entirely by changes in F2 for the male continuum, it was perceptually less salient.

A final possibility is that outcomes for the male speaker continuum were influenced by the fixed order in which the continua were presented. Because the male speaker continuum was presented at the end of the experiment, when participants were more likely to be fatigued, it could be that some children’s performance on the male speaker task was influenced more by attentional issues than by perceptual acuity. The fact that age was a significant predictor of performance on the male speaker continuum but not the female speaker continuum is consistent with this possibility, as younger children could be expected to experience greater difficulty sustaining attention. There could also be an interaction between these two experimental factors (i.e., the differences between the continua and the order effect). Suppose that, as posited here, the stimuli from the male speaker continuum are intrinsically easier to classify, but the order effect makes the task attentionally demanding. Children who can sustain attention to that point in the experiment should then perform very well, while children who have not sustained attention will fare poorly; this tells us more about age and attentional capacities than it does about perceptual acuity. A version of the experiment in which the male stimulus continuum is presented first would be necessary to test this hypothesis.

### Future directions

Although a significant perception-production correlation was predicted in the present case, it was not a foregone conclusion, since previous research has documented dissociations between perception and production abilities in the course of development. In their study of children aged 4–8.5 years, Idemaru and Holt [40] did not find a correlation across individuals between the weight of an acoustic cue, F2, in perception and production. Idemaru and Holt [40] suggested that child-specific articulatory limitations might account for their finding: some child speakers with a well-specified perceptual target could still lack the articulatory skill to achieve the complex tongue configuration associated with /ɹ/. The older children in the present study can be presumed to represent a more advanced stage of development at which gross articulatory limitations have receded, allowing individual differences in the specificity of perceptual targets (e.g., [2]) to manifest in parallel differences in production. Additional research on children in an intermediate age range (e.g., 6–10 years) could illuminate the point at which the hypothesized limitations are overcome. Articulatory as well as acoustic measures will play an important role in understanding this developmental trajectory.

One additional implication of this study pertains to the development of synthetic speech continua for use in research and clinical intervention. In the present study, identical methods were used to develop two synthetic continua from *rake* to *wake*. However, one of the two continua was judged to be more successful than the other in assessing perceptual sensitivity for /ɹ/. As reported above, both continua were characterized by a steady rise in F3-F2 distance from the *rake* to the *wake* endpoint. In the female continuum, this was achieved by incremental changes to both F2 and F3, but in the male speaker continuum, lowering of F2 played a much larger role than raising of F3. This difference may have contributed to the distinct patterns of response to the continua that we observed in both child and adult listeners. Note that the resynthesis technique used here was not intended to selectively manipulate the primary and secondary cues of F2 and F3 height; this difference between the continua is an artifact. As a result, we do not attempt to interpret these results in terms of cue weighting. However, follow-up research systematically investigating the relative contributions of these two cues is an essential direction for follow-up research.

As a practical matter, the present finding that experimental outcomes can be highly sensitive to the properties of experimental stimuli reinforces the importance of norming or at least extensively piloting perceptual stimulus materials prior to experimental data collection. The crowdsourced rating method used for post-hoc testing in the present study represents a rapid and convenient means to evaluate the properties of speech continua. Lastly, our experience in this study provides an argument in favor of sharing stimulus materials whose properties have been established in previous testing. The continua used in this experiment are available as an electronic supplement to this paper (S1 and S2 Files); researchers are particularly encouraged to use the female speaker continuum (S1 File), which was found here to provide a reasonably sensitive measure of rhotic perception.

## Conclusions

This study measured perception and production of rhotic sounds in typically developing speakers aged 9–14. Across participants, there was a significant correlation between rhoticity in production and perceptual acuity as measured with one of two continua. No significant correlation between perception and production performance was found for a second continuum. This was hypothesized to reflect differences between the stimulus sets, but it could instead be interpreted as an indication that the correlation observed in the context of the female speaker continuum is not a robust finding. These results can be considered preliminary evidence that children with a more refined auditory target for a sound also produce that sound more accurately. However, a refined and higher-powered study will be necessary to draw strong conclusions.

The significant correlation reported here is consistent with previous literature documenting systematic relations between perceptual acuity and production distinctness for various contrasts in both children and adults (e.g., [2, 4, 5, 27]). This finding is compatible with the idea of auditory targets for speech production (e.g., [8]): that is, speakers who specify a narrow target range for rhotic sounds also tend to realize rhotics with more extreme acoustic properties.

Finally, the results of this study may have implications for the clinical management of speech delay and disorder in children. The present findings suggest that some of the normal variation in children's production of rhotics can be attributed to perceptual rather than articulatory differences. This supports the hypothesis, suggested in previous research (e.g., [47, 48]) that at least some children with rhotic misarticulation have a core deficit in their auditory-perceptual specification of the target for /ɹ/. Further research should collect fine-grained perceptual measures from children, adolescents, and adults who present with derhotacized production of /ɹ/. It is possible that perceptual measures will have prognostic value in predicting the persistence of rhotic errors. They may also have implications for intervention practices, e.g. by supporting an expanded emphasis on perceptual training in treatment targeting rhotics. Additional detail about the relative roles of speech-motor and perceptual development in children with residual rhotic errors can be hoped to lead to improved interventions customized to an individual’s specific profile of abilities and deficits.

## Supporting information

S1 File Sound file containing all ten steps of the *rake*-*wake* continuum synthesized from the ten-year-old female speaker. (WAV)

S2 File Sound file containing all ten steps of the *rake*-*wake* continuum synthesized from the eight-year-old male speaker. (WAV)
